# Supplementary material for: Are teachers meeting students’ needs in untracked science classrooms? Evidence based on a causal inferential approach
Source: PLoS One. 2024 Apr 16;19(4):e0300587. doi: 10.1371/journal.pone.0300587 (PMC11020409; doi:10.1371/journal.pone.0300587)

**S1 Appendix: Descriptive statistics**

The correlations between adaptive instruction and personal feedback, adaptive instruction and emotional support, and personal feedback and emotional support are 0.58, 0.56, and 0.45 respectively, suggesting that the constructs are related yet distinct enough to treat as separate outcomes in our analyses. These correlations are similar within the other subgroups of tracked students, untracked students, students of the same ethnicity, and students of similar economic-social-cultural status (ESCS), suggesting that these constructs could be treated as separate outcomes in those subgroups as well.

The descriptive statistics of the three outcomes of interest are also presented in Table S1-1 for all students and for certain subgroups. The subgroups of interest, which correspond to those traditionally underserved by tracking, are minority students (Black or Hispanic students), low-income students (students whose ESCS, or economic-socio-cultural statuses, are below the PISA average), and immigrant students (first-generation immigrants). However, the latter is not included in Table S1-1 because of small sample size – there are only 45 first-generation immigrant students in the sample (comprising about 5% of the total sample). For comparison, descriptive statistics for White and Asian students as well as above average ESCS students are also provided.

The preliminary results for the entire sample of students suggest that there may not be such large differences in perceptions of teaching between the two types of schools. The results for the various subgroup are slightly more mixed – most outcomes are roughly equal while a few are not. For example, while Black and Hispanic students reported similar levels of adaptation of instruction (adaptive instruction) and provision of teacher support (emotional support) across the different types of schools (a result which also holds for the White and Asian students), above-average ESCS students reported greater levels of personal feedback in tracked schools. Across all the subgroups and outcomes, personal feedback appears to have the largest disparities across the different types of schools. However, it is difficult to draw definitive conclusions from the small sample sizes of these subgroups (see Table S1-2 for the sample sizes and proportions for those subgroups in the analytic sample). Due to this limitation, we focus throughout this paper on the sample of all students.

Finally, we were curious to see whether students between the tracked and untracked schools may differ in their motivations. To that end, in Figure S1-1, we consider the distributions of the “MOTIVAT” index variable, described in the PISA 2015 Technical Manual as an index derived from students’ agreement with the following statements: I want top grades in most or all of my courses; I want to be able to select from among the best opportunities available when I graduate; I want to be the best, whatever I do; I see myself as an ambitious person; I want to be one of the best students in my class. Higher values indicate students have greater academic motivation. The distributions look similar, suggesting that in our sample, students do not vary significantly between the two types of schools and also suggesting that we need not worry about this potential confounder.

**Table S1-1**

*Means and standard deviations of outcomes for different student subgroups*

|  | All students | | Black and Hispanic students | | White and Asian students | | Below average ESCS students | | Above average ESCS students | |
| --- | --- | --- | --- | --- | --- | --- | --- | --- | --- | --- |
| Outcome | Tracked | Untracked | Tracked | Untracked | Tracked | Untracked | Tracked | Untracked | Tracked | Untracked |
| Adaptive instruction | 0.25 (0.97) | 0.23 (1.00) | 0.20 (1.02) | 0.20 (1.06) | 0.27 (0.93) | 0.26 (0.98) | 0.29 (0.95) | 0.17 (1.03) | 0.23 (0.98) | 0.30 (0.98) |
| Emotional support | 0.37 (0.94) | 0.34 (0.99) | 0.37 (1.00) | 0.33 (1.01) | 0.36 (0.91) | 0.33 (0.98) | 0.33 (0.96) | 0.31 (1.03) | 0.39 (0.93) | 0.36 (0.96) |
| Personal feedback | 0.39 (1.10) | 0.32 (1.12) | 0.52 (1.17) | 0.42 (1.06) | 0.32 (1.04) | 0.27 (1.17) | 0.48 (1.10) | 0.38 (1.11) | 0.34 (1.10) | 0.26 (1.13) |

*Note.* Outcome measures are standardized with a mean of 0 and a standard deviation of 1.

**Table S1-2**

*Frequency counts and proportions of student subgroups by tracked or untracked schools*

| Student demographic | Untracked school | Tracked school |
| --- | --- | --- |
| Black or Hispanic | 218 (37.7%) | 131 (39.5%) |
| White or Asian | 320 (55.4%) | 188 (56.6%) |
| Below average ESCS | 243 (42.0%) | 120 (36.1%) |
| Above average ESCS | 335 (58.0%) | 212 (64.0%) |

*Note*. Proportions denote the percentage of students in the given school type that comprise the given demographic.

**Figure S1-1**

*Difference in distributions of the MOTIVAT index variable between students in tracked and untracked schools*


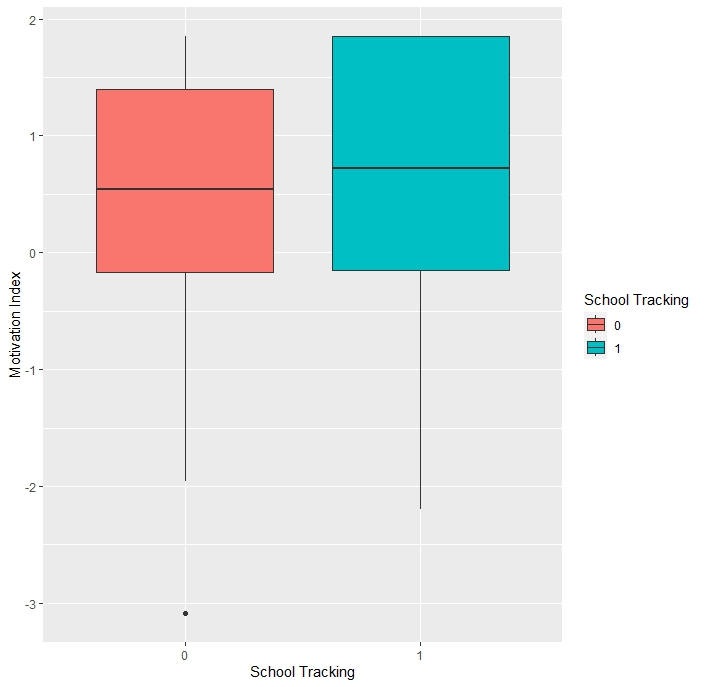

Supplement: S1 Appendix — We provide descriptive statistics between tracked and untracked schools and among different student demographics. (DOCX) [file pone.0300587.s001.docx]
