# Supplementary material for: Are teachers meeting students’ needs in untracked science classrooms? Evidence based on a causal inferential approach
Source: PLoS One. 2024 Apr 16;19(4):e0300587. doi: 10.1371/journal.pone.0300587 (PMC11020409; doi:10.1371/journal.pone.0300587)
Supplement: S2 Appendix — We provide descriptive statistics of the outcomes between tracked and untracked schools and among different student demographics. (DOCX) [file pone.0300587.s002.docx]

**S2 Appendix: Missing data problem**

One concern is that the exclusion of cases with missing data may bias our results – even before trimming, students or schools had to be excluded because of missing data on covariates or outcomes. To evaluate whether the data may be missing not at random (MNAR), the means of the covariates between the full data (which includes cases with missing outcome measures) and the trimmed data were compared in Table S2-1. Although it is not possible to definitively prove whether the data is MNAR, the comparison suggests that the means of the covariates are rather similar between the two sets.

**Table S2-1**

*Means of covariates between full data and data with missing values removed*

| Covariate | Full data | Missing values removed |
| --- | --- | --- |
| Percentage of students receiving free and reduced price lunch | 3.58 | 3.35 |
| Student-teacher ratio | 15.63 | 15.49 |
| Percentage of 10^th^ graders who are non-native English speakers | 25.59 | 22.79 |
| Percentage of 10^th^ graders from SES-disadvantaged homes | 15.57 | 15.94 |
| Percentage of 10^th^ grade students enrolled in special education programs | 50.55 | 44.28 |
| Resources available for science instruction | 5.71 | 6.18 |
| Levels of economic-socio-cultural status | 0.02 | 0.15 |
