# Supplementary material for: Are teachers meeting students’ needs in untracked science classrooms? Evidence based on a causal inferential approach
Source: PLoS One. 2024 Apr 16;19(4):e0300587. doi: 10.1371/journal.pone.0300587 (PMC11020409; doi:10.1371/journal.pone.0300587)

**S3 Appendix: Sensitivity analyses**

The first part of our sensitivity analysis compares the results we attained with those we would have gotten had we utilized other more naïve approaches. The first approach is the difference-in-means approach, in which the difference in outcomes between the groups without any adjustments for self-selection or for survey weights is taken. The second approach is the stratification approach in which the treatment effects are estimated using the propensity score strata without incorporating the survey weights or MMW. The purpose of this is to investigate whether the aforementioned results seem reasonable, following the suggestion of Schafer and Kang (2008).

The second part of the sensitivity analyses was conducted using the approach and the sensemakr R package provided by Cinelli and Hazlett (2020). We use this approach and package because their framework allows us to argue the plausibility of unaccounted confounders that could change our results. Their software (Cinelli et al., 2020) computes the robustness values and other sensitivity analyses statistics as derived in their paper. Robustness values *RV_q_* are the amount of confounding necessary to reduce or increase the estimated treatment effect by (100 × *q*)% to an unacceptable level. *RV_q_* is related to the partial *R^2^* of the outcome *Y* with the hypothetical confounder *Z* given the covariates *X* and treatment indicator *D* included in the model (i.e. *R^2^_Y~Z|X,D_*) and the partial *R^2^* of the treatment indicator *D* with the hypothetical confounder *Z* given the covariates *X* (i.e. *R^2^_D~Z|X_*) such that *RV_q_* = *R^2^_Y~Z|X,D_* = *R^2^_D~Z|X_*. In other words, these residual variances of the outcome variable and treatment variable determine the effect of the unobserved confounder(s) on the estimated treatment estimates. See Cinelli and Hazlett (2020) for detailed explanations of these quantities.

Finally, to make sense of the robustness values, as mentioned earlier we chose the benchmark covariates of SCIERES and FRPL to evaluate how likely it is that we have omitted a covariate important enough to affect our results. These benchmark covariates are thought to be our strongest confounders, with strong associations with the outcome and treatment. We can compare these associations with the robustness values to see how likely it is that a hypothetical unobserved confounder, as strongly associated with the treatment and the outcome as the benchmark covariate (or several times more strongly associated), could exist.

**Data aggregation and modified sample in the sensitivity analyses**

Because the sensemakr package only works with ordinary least-squares regression and the treatment is nominally at the school-level, the student-level outcomes were aggregated to school-level means. Whereas we had analyzed data at the student-level in the first part of the analyses, we had to aggregate all information to the school-level to utilize this software for sensitivity analyses. The aggregated school-level means were then standardized to have zero means and unit variances across schools in order to facilitate interpretation of the effect size estimates in our regression models in which the student-level outcomes, now aggregated to the school-level, were regressed on school-level covariates and the treatment indicator variable. This regression model was then assessed using the sensemakr package, which also provided the robustness values.

Since all measures were aggregated to the school-level, the effective sample size was reduced to the number of schools. To attenuate small sample size problems, we used all three groups, with groups A and B (students in schools that tracked by all subjects and by some subjects, respectively) being aggregated to create a new, larger tracked (treatment) group. Considering the prevalence of honors, International Baccalaureate, and Advanced Placement courses in biology, chemistry, and physics at U.S. high schools, and the fact that science is a similar subject to core subjects like English and math which are commonly tracked, we assumed that the vast majority of schools that engage in “some” tracking (group B) do so in science classes even though the data do not include details on exactly which subjects are tracked at those schools. This assumption is further supported by the fact that about 78% of schools in the base year data of the Educational Longitudinal Study of 2002 (ELS:2002) reported that they track (whether by differentiated grouping or enforcing required course prerequisites for differentiated courses) for courses in the core curriculum, defined to be courses in English, math, science, and social studies. Since the ELS and PISA U.S. samples are supposed to be nationally representative of schools, this data suggests that if U.S. schools were to track for some subjects, science (and other subjects in the core curriculum) would be one of those subjects. We thus argue that treating groups A and B as schools that track by science is a defensible and plausible assumption.

**Comparisons with other approaches**

The treatment estimates derived from a naïve difference-in-means estimate (DIM) between the treatment and control groups (i.e. taking the difference in outcomes between the groups without any adjustments for self-selection or for survey weights), from using the propensity score strata created from MatchIt (Stratification) without incorporating the survey weights or MMW, and from the combination of the final weights and multilevel model (Multilevel) were compared. For adaptive instruction, the treatment effects were DIM = -0.03, Stratification = 0.01, Multilevel = -0.02; for personal feedback, DIM = 0.04, Stratification = 0.08, Multilevel = 0.06; for emotional support, DIM = 0.04, Stratification = 0.05, Multilevel = 0.04. The results overall seem consistent across these different approaches, suggesting that our findings are robust to differences in the approach used.

**Robustness to unobserved confounding**

Because the results from the causal inferential analysis suggest a null treatment effect, the ensuing analyses provide the robustness values that would increase our treatment estimates to an unacceptable level, which we define using Cohen’s effect sizes (Cohen, 1969). One of the key arguments made in favor of tracking is that teachers can be more responsive to student needs in classes where ability levels are more homogeneous, so the null hypothesis of our sensitivity analysis considered biases that would increase the values of the effect estimates to a level that would be both substantively meaningful in magnitude and positive. Specifically, we considered unobserved confounding that would increase the effect sizes to +0.2 standard deviation, a widely used threshold for effects in the “small” to “medium” range in social science research (Cohen, 1969). This seemed reasonable because our treatment effect sizes are in the “small” range, so we would want to know how much unobserved confounding would need to exist to move our effect sizes to the next defined range.

Table S3-1 shows the estimated effects of tracking on each of the three outcomes of interest (thereby corroborating our prior results, albeit with a different approach) along with the standard errors, t-values, and the robustness values. The magnitudes of the point estimates were negligible (all within one-tenth of a standard deviation) and not statistically significant. As we found in our earlier analyses, we did not find evidence to support tracking proponents’ claims that grouping students by ability has a substantial positive effect on how well science teachers adapt instruction, provide adequate feedback, or provide individualized support.

The robustness values appear in the rightmost column. (We use the notation *RV_q_* to denote the robustness value associated with the amount of unobserved confounding that would be necessary to increase the point estimate of the standardized treatment effect to +0.2.) These robustness values imply that to bring the treatment effect point estimate to 0.2, unobserved confounders would need to explain more than

• 6.4% of the residual variance of both school tracking and adaptation of instruction;

• 8.6% of the residual variance of both school tracking and provision of adequate feedback; and

• 9.7% of the residual variance of both school tracking and provision of individualized support.

To better interpret these robustness values, it is helpful to think of hypothetical confounders that could exist to increase the point estimates to an unacceptable amount. To assess whether unobserved confounders strong enough to change the treatment effect point estimates to +0.2 might plausibly exist, we selected two of our observed pretreatment covariates—resources available for science instruction (SCIERES) and school mean economic-socio-cultural-status (ESCS)—as benchmark covariates. We chose these covariates as benchmarks because they have the strongest association with the outcomes among all of the covariates included in our OLS models and because they are the two strongest predictors of treatment assignment when we regressed the treatment indicator on the observed covariates. Moreover, as discussed earlier, extensive prior research provides a theoretical basis for how and why the student and school characteristics captured by SCIERES and ESCS would positively influence selection into treatment as well as the outcomes (Delpit, 1995; Domina et al., 2016; Gay & Howard, 2000; Hallinan, 2004; Lareau, 2011; Loveless, 2016; Oakes, 2005).

The sensitivity contour plots in Figure S3-1 are visual representations of how the effect size estimates would change in the event of unobserved confounding five times as strong as the benchmark covariates. The red dotted contour denotes the combinations of associations of the hypothetical confounder (in terms of *R^2^*) with the treatment and outcome at which the treatment effect estimate (marked with the black triangle in the lower left corner in each graph) would reach the threshold of +0.2 standard deviations. The smaller red diamonds denote hypothetical unobserved covariates which have five times the confounding associations as the benchmark covariates. Since the red plots are not generally close to the threshold, even unobserved confounders five times as strong as the two benchmark covariates would not be able to drive our effect estimates to the problematic threshold. We can see that unobserved confounding would need to be considerably stronger than five times as strong as our benchmark covariates to move the point estimates into the “small-to-medium” effect size range of 0.2 or greater. Given that we believe that SCIERES and ESCS are the strongest possible confounders, we find it unlikely that unobserved confounders more than five times as strong as SCIERES or ESCS could exist to overturn our results.

**Table S3-1**

*Effect size estimates and robustness values*

| Outcome | Estimate | Standard Error | t-value | *RV_q_* |
| --- | --- | --- | --- | --- |
| Adaptive instruction | 0.031 | 0.227 | -0.745 | 6.4% |
| Personal feedback | -0.012 | 0.209 | -1.017 | 8.6% |
| Emotional support | -0.063 | 0.228 | -1.151 | 9.7% |

*Note.* df = 128

**Figure S3-1**

*Contour plots of sensitivity analyses for the outcomes*


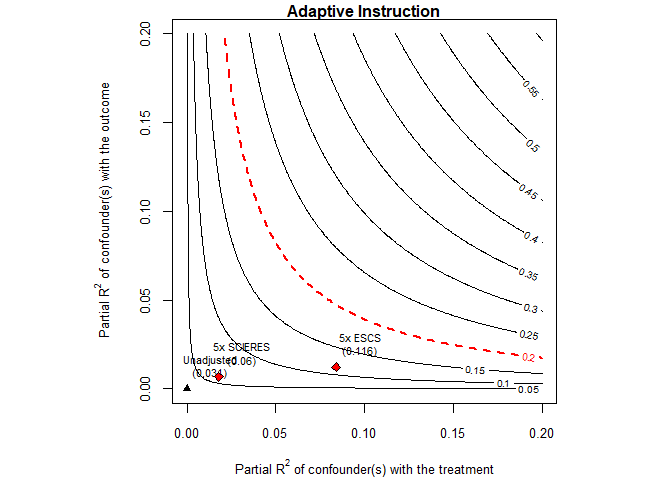


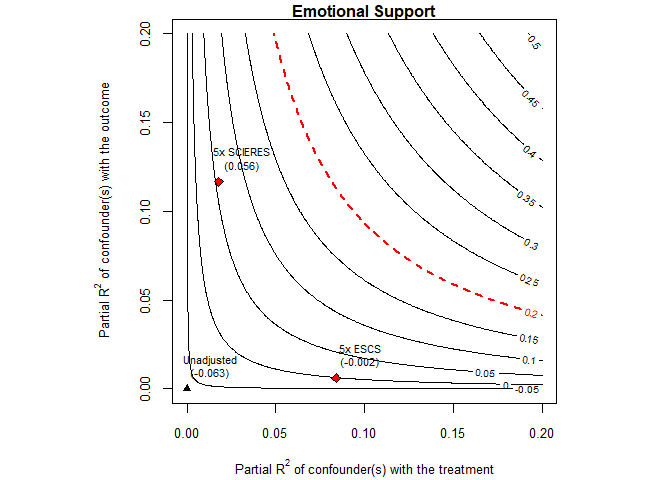

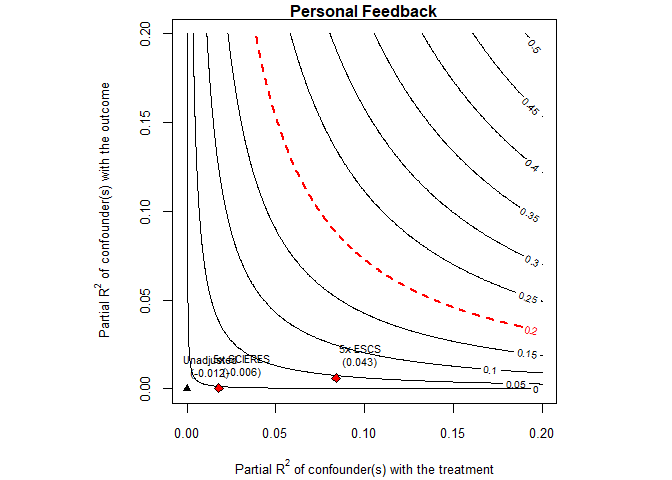

Supplement: S3 Appendix — We conduct sensitivity analyses to evaluate whether our results seem reasonable and robust to unobserved confounding. (DOCX) [file pone.0300587.s003.docx]
