# Supplementary material for: Are teachers meeting students’ needs in untracked science classrooms? Evidence based on a causal inferential approach
Source: PLoS One. 2024 Apr 16;19(4):e0300587. doi: 10.1371/journal.pone.0300587 (PMC11020409; doi:10.1371/journal.pone.0300587)
Supplement: Attachment — Submitted filename: Response to Reviewers.docx [file pone.0300587.s004.docx]

Dear Academic Editor and Reviewers:

Thank you for your thoughtful feedback and comments regarding our manuscript entitled *Are teachers meeting students’ needs in untracked science classrooms? Evidence based on a causal inferential approach* (PONE-D-23-30374). Below, we have explained the changes we have made to address your comments. These changes are highlighted in the revised version of the manuscript.

**Reviewer 1 Comments**

*1- The findings have some limitations mainly due to the limited sample size.*

*2- The standard errors of effect size estimates were fairly large*

*possibly due to the small sample size.*

*3- The study focuses on American schools.*

*4- The authors have the option to publish the results from this study in a more appropriate journal. The comments 1 to 3 suggest that these results only provide preliminary evidence that detracking may be a promising educational intervention that does not negatively affect students’ perceptions of their teachers' ability to meet their students' needs.*

Thank you for your comments and suggestions. Comments 1 through 3 were mentioned in the “Limitations” section. In the “Discussion” section, we have also noted that our results are only preliminary evidence regarding the promise of detracking. Finally, the authors have collectively agreed that *PLOS ONE* would be a suitable outlet for our study.

*5- There are any grammatical errors should be corrected at revision.*

*For example (in abstract):*

*"Tracking has been criticized........ which students encounter greater lack of instructional support."*

*I suggest:*

*"Tracking has been criticized........ which students encounter a greater lack of instructional support."*

*Other example:*

*Line 566: The results from this study have*

*Line 567: strong implications and significance for policymakers and educators .........*

*I suggest:*

*Line 566: The results of this study have*

*Line 567: significant implications for policymakers and educators .........*

Thank you for your comments. We have edited the text as you have suggested.

**Reviewer 2 Comments**

*1. Evidence based on a causal inferential approach is currently applied in the educational and social settings. Moreover, it is one of the most successful epistemological breakthroughs in the field of post-pandemic research today.*

*2. Well, according to the logic of deductive inference, the data provided by the study support the argument used. This argument in turn guarantees and facilitates the understanding of the fact or phenomenon under study.*

Thank you for your support of our manuscript.

Please do not hesitate to contact us for more information. Thank you again for your consideration of our manuscript.

Best regards,

The authors
